# Miniaturized paper-based analytical device for the portable analysis of phyto-cannabinoids in plant and oral fluids

**DOI:** 10.1007/s00216-023-05013-x

**Published:** 2023-11-04

**Authors:** Dymphy Houtzager, Sergio Armenta, José Manuel Herrero-Martínez, Héctor Martínez-Pérez-Cejuela

**Affiliations:** Department of Analytical Chemistry, University of Valencia, Dr. Moliner 50, 46100 Burjassot, Valencia Spain

**Keywords:** Cannabis, Paper-based device, Colorimetry, Phyto-cannabinoids, On-site analysis

## Abstract

In this work, a low-cost and eco-friendly paper-based analytical device (PAD) method is described for the determination of phyto-cannabinoids in cannabis and oral fluids based on a simple colorimetric reaction. The PAD was able to distinguish tetrahydrocannabinol (THC)- and cannabidiol (CBD)-rich plant samples by using 4-aminophenol (4-AP) and later on to quantify total phyto-cannabinoid content (THC + CBD + CBN) in plant and oral fluids by using the Fast Corinth V reagent. The chemical and physical properties regarding paper type and reagent concentration in the PAD were optimized to achieve the best analytical performance. After that, analytical features were obtained, including a linear range of 0.01–0.1 mg mL^−1^, a limit of detection (LOD) of 0.003 mg mL^−1^, and a suitable precision, expressed as relative standard deviation (RSD) lower than 10%. Furthermore, no significant interferences were observed in colorimetric reactions when tea, herbs, and drug samples were analyzed. Additionally, the PAD proved color stability up to 1 month after the sampling at 25 °C. The developed PAD was suitable for determining total phyto-cannabinoid content in plants and oral fluids, obtaining good results compared to GC–MS. Overall, this method showed good reliability resulting in an operational on-site device for drug monitoring.

**Graphical Abstract:**

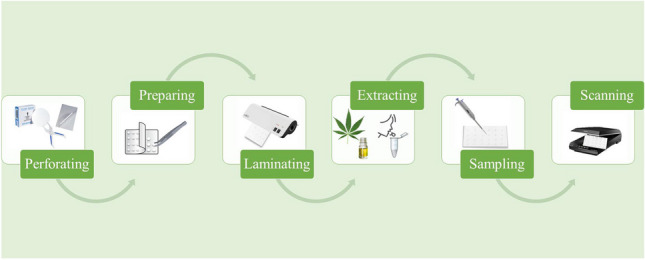

**Supplementary Information:**

The online version contains supplementary material available at 10.1007/s00216-023-05013-x.

## Introduction

**Fig. 1:**
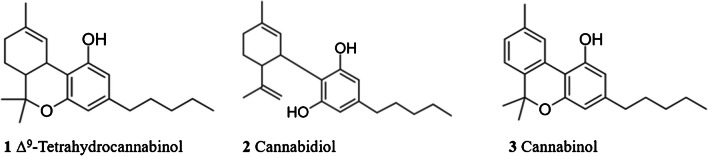
Chemical structures of Δ^9^-tetrahydrocannabinol, cannabidiol, and cannabinol

Cannabis, also called marijuana, is the most widely used illegal drug worldwide and it is consumed for recreational, entheogenic, and medicinal purposes. The 2022 World Drug Report of the United Nations Office on Drug and Crime office (UNODC) stated that around 4% of the global population aged 15–64 consumed cannabis in 2020 [1, 2]. Cannabis consists of more than 400 chemical compounds, 60 of which are identified as phyto-cannabinoids [3]. These compounds are the active chemical substances produced by the plants *Cannabis sativa* and *Cannabis indica,* being Δ^9^‐tetrahydrocannabinol (**1** Δ^9^‐THC), the primary psychoactive phyto-cannabinoid. Cannabidiol (**2** CBD) and cannabinol (**3** CBN) are also significantly present in cannabis, being not psychoactive components [4] (see Fig. 1). Cannabis plants can be categorized as hemp or marijuana, whereby hemp contains a Δ^9^-THC concentration lower than 0.3% wt. and marijuana a Δ^9^-THC concentration higher than 0.3% wt. [5]. Furthermore, the European Union has a maximum allowed Δ^9^-THC content for products derived from cannabis of 0.3% wt. [6].

UNODC recommended different analytical techniques for the analysis of cannabis products, including colorimetric tests, immunoassays, ion mobility spectrometry, and chromatography-based methods [7]. However, most of them require benchtop equipment. Others, such as thin-layer chromatography (TLC) or infrared spectroscopy attempt to overcome the high purchase cost as well as the simplicity in the operation [8]; however, there are no reports of using a single technique to both qualify and quantify in a straightforward manner.

For this reason, an interesting alternative to cannabis identification could be the colorimetric assays (rapid tests) due to their portability and cost-efficiency. Among the different available colorimetric reagents, UNODC recommends the use of Fast Corinth V salt, Fast Blue B salt, and the Duquenois-Levine procedures [7]. The Fast Blue B reagent has previously been used to differentiate between hemp-type and marijuana-type cannabis [8] as well as quantifying the total phyto-cannabinoid content in cannabis samples [9]. It was reported that Fast Blue B forms a red chromophore in the presence of THC, and an orange chromophore in the presence of CBD. Another colorimetric test currently being used to differentiate between hemp and marijuana is the 4-aminophenol (4-AP) test, which has been developed by the Swiss Forensic Institute in Zurich [10]. The 4-AP reaction provides a pink color when the THC/CBD ratio is below 0.3 (CBD-rich), and a blue color when the ratio of THC/CBD is above 3 (THC-rich) [11]. The possibility to perform such colorimetric tests on paper-based analytical devices (PADs) will provide portability, use of low amount of reagents, ease to use, reduced waste generation, low cost, and high throughput capabilities. However, only one report can be found in the bibliography that couples PAD technology with colorimetric reaction devoted to analyse phyto-cannabinoids in real samples [13]. For this reason, the application of PADs for cannabis testing can be a realistic and attractive alternative not only for laboratory, but also for *in-field* settings.

In summary, this work represents the first low-cost (€0.10 per PAD) and simple (only requires stationery material) PAD approach for portable phyto-cannabinoid monitoring in terms of qualification and quantification in real samples. For this purpose, 4-AP colorimetric reaction discriminates between hemp-type and marijuana-type cannabis samples, and the total phyto-cannabinoid content will be determined using the Fast Corinth V colorimetric test. The PAD design minimizes both waste generation and cross-contamination since the different reaction zones (24 per PAD) are separated by a sealed laminating pouch (physical barrier of approx. 1 cm of plastic). The method was optimized, including the chemical part (reagents concentration and volumes) and the physical part, such as paper type, thickness, pore size, etc. Finally, the optimized protocol was validated and applied to qualify THC/CBD in plant samples and to quantify phyto-cannabinoids in plant and oral fluids.

## Experimental

### Reagents, samples, and solutions

Analytical-grade chemicals and Milli-Q water (MQW) (resistivity > 18 M cm^−1^, Millipore, Bedford, MA, USA) were used to produce all solutions.

The 4-aminophenol (4-AP) assays were performed using the 4-AP reagent (reagent A) and sodium hydroxide (reagent B). The 4-AP reagent was prepared by weighing 30 mg 4-AP (≥ 98%, Sigma Aldrich) and dissolving in 99.5 mL ethanol and 0.5 mL of 2 M hydrochloric acid. The sodium hydroxide was prepared by weighing 3 g of sodium hydroxide in 30 mL of MQW and 70 mL of ethanol. The 4-AP reagents were stable in amber bottles at 8 °C for 6 months.

The Fast Corinth V reagent (Cannabis Special Solid Reagent, VWR chemicals) was prepared with 10 mg of reagent in 1 mL of MQW to give a final concentration of 10 mg mL^−1^. The colorimetric reagent was prepared weekly by proper dilution with MQW and stored at -20 °C to avoid degradation.

A Δ^8^-THC, Δ^9^-THC, CBD, and CBN stock solution (1 mg mL^−1^, Sigma Aldrich) was used for the preparation of working standards, and proper dilutions were done with methanol.

A Δ^9^-THC-D3 stock solution (0.1 mg mL^−1^) was used as an internal standard (IS) for the GC–MS confirmation method.

Cannabis samples were kindly provided by the Pharmaceutical Inspection and Drug Control Unit of the Health Service Area of the Spanish Government in Valencia. Oral fluids were obtained from volunteers who, after appropriate information, consented to provide samples following the Ethical guidelines established by the Universitat de València (H1454687358321 Drug analysis in biofluids). Volunteers were males and females aged from 18 to 45 years old and none of them were currently using cannabis or hemp products, except for the individual mentioned in Table 3, who declared as a cannabis consumer. Samples were collected in an Eppendorf tube 1.5 mL of capacity and stored at -20 °C until analysis.

### Sample preparation

Different cannabis samples were analyzed by extracting 25 mg of dried cannabis samples with 2 mL of methanol. For this, the mixture was stirred for 5 min, and then, the mixture was separated by gravity. The resulting supernatant was diluted 40-fold times with MeOH before being analyzed.

Regarding oral fluid samples, they were processed by liquid–liquid microextraction (LLME). Briefly, 1 mL of sample was extracted with 25 µL chloroform (to achieve a 40-fold enrichment factor), then mixed on a shaker for 5 min and centrifuged for 3 min at 14,000 g force. The organic phase was collected with the help of a micro syringe, placed in a 2 mL glass vial, and stored in the freezer until analysis.

### PAD fabrication

**Fig. 2:**
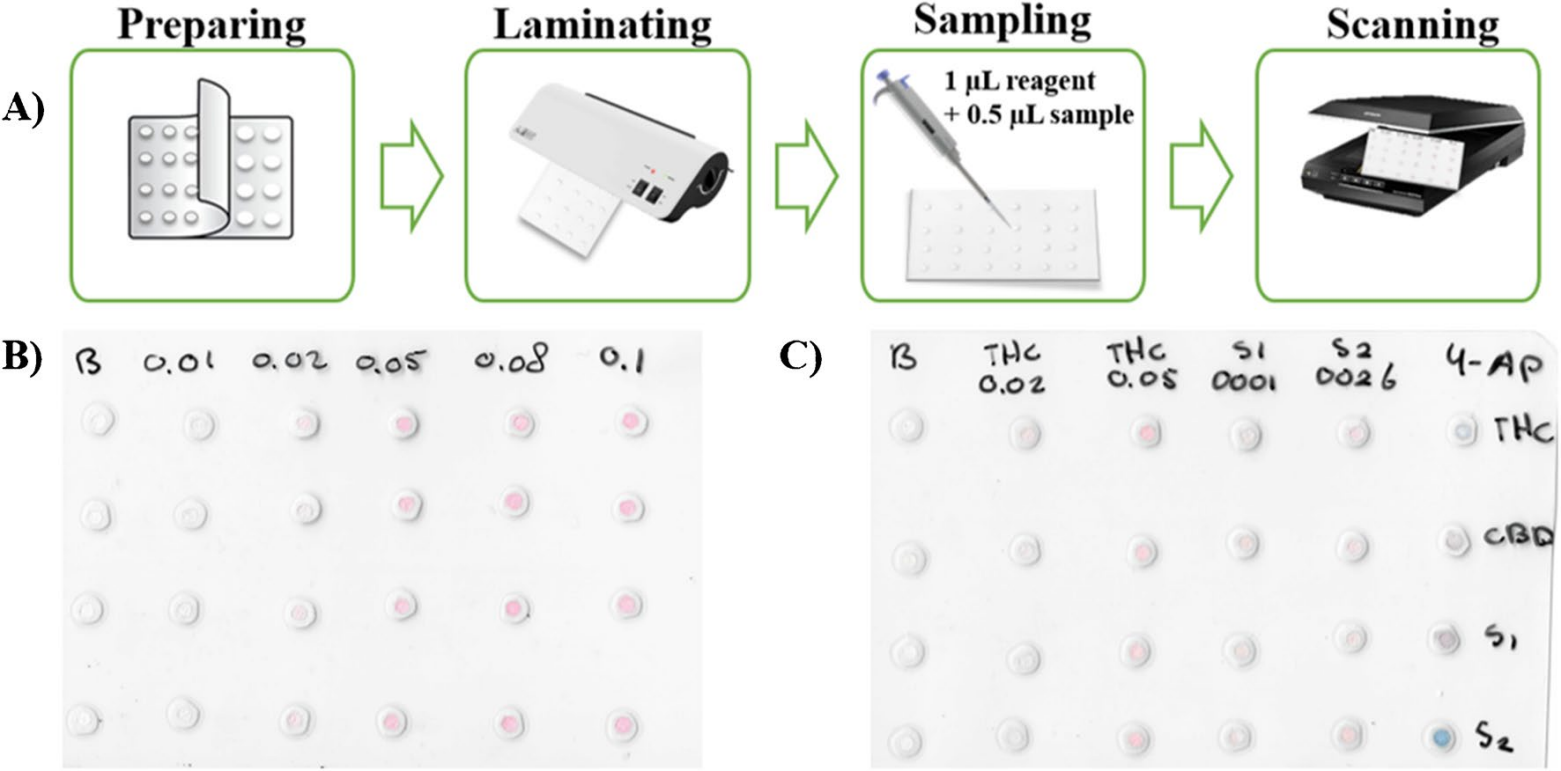
**A**) Schematic representation of a PAD assembly and operating method. **B**) Example of accurate PAD scanning capture using the Fast Corinth V reagent following the loading of standards and a 15-min wait and **C**) the final PAD design with two cannabis samples, 4-AP reagent for the discrimination of CBD/THC and quantification of phyto-cannabinoids using the Fast Corinth V reagent and Δ^8^-THC standards

The PAD device was performed with simple stationery material. Its construction consisted of a 4 × 6 units distribution (columns x rows) with 24 reaction units (see Fig. 2A for PAD assembly procedure). The hydrophilic zones were based on paper discs, meanwhile the hydrophobic zones were produced by a melted laminating pouch. In the assembly process, the paper (Whatman® nº 1) was previously cut to 4 mm with a puncher (400/3 to 20 IRAZOLA BAHCO), and the laminating pouch perforated with 2 mm holes (Knipex puncher, Wuppertal, Germany). Then, all the discs were equidistantly placed one by one between the 2 layers in the plastic pouches (75 × 110 × 0.125 mm, Q-Connect, Gent, Belgium), which could be time-consuming if many layers are required. Next, the plastic was melted by a heating process with a benchtop laminator (United Office, Cleveland, USA), and a single-layer device was obtained. After this process, the PAD card was ready to use.

### Analysis with PAD and data treatment

#### Cannabis type identification

For the discrimination of the type of cannabis samples (CBD-rich, THC-rich plants), the 4-AP colorimetric reagent was used. For this purpose, the reagents were added in the following amounts: 1 µL of reagent A (4-AP), 1 µL of sample, and 0.5 µL of reagent B (sodium hydroxide) into the PAD. Under the basic conditions of reagent B, reagent A will be converted into 4-iminoquinone, which will then react with the free-para-phenol positions in the phyto-cannabinoids. This results in structures with different degrees of conjugation and, thus different colors [11]. After 5 min, a pink color was developed when the sample was CBD-rich, and a blue color appeared when the sample had a high content of THC/CBN.

#### Total phyto-cannabinoid quantification (THC + CBD + CBN)

For the quantification of the phyto-cannabinoid content (Δ^8^-THC, Δ^9^-THC, CBD, CBN) in plants, the following procedure was used. First, 1 µL of the Fast Corinth V reagent was introduced, and subsequently, 0.5 µL of standard/sample extract was loaded onto the hole placed over the detection layer. For the quantification of the phyto-cannabinoids in oral fluids the following guidelines were followed: 1 µL of reagent was firstly added, and then, 1 µL of the sample extract was loaded onto the PAD. The phyto-cannabinoids react with the reagent forming a pinkish color (see Fig. 1B). The intensity of the pink color is directly proportional to the concentration of phyto-cannabinoids in the sample. After 15 min at room temperature (20–25 °C), the volume was entirely absorbed, and the pink color developed. The images (.png format) were captured with a scanner (Epson) and processed using the free software ImageJ® (105 × 105 pixels) by using a green filter. The intensity of the signal was established using 4 replicates per measurement (blank/standard) and removing potential outliers, if necessary. The intensity values were converted to pseudo-absorbance following the expression: A = log (I_0_/I), where I is the standard/sample intensity, and I_0_ is the blank intensity obtained by loading methanol. Then, the samples were quantified using calibration curves with the calculated pseudo-absorbance for each standard and the corresponding Δ^8^-THC concentration (see Fig. 2B and C for final PAD). When sample has been classified as CBD-rich, the total amount of phyto-cannabinoids will be obtained multiplying the obtained value by a factor of 2.2 to compensate the different sensitivity of CBD and THC in colorimetric reaction of the device. This normalization factor allows the use of THC calibration curve for any kind of sample, simplifying the operational work.

### Reference procedure—GC–MS

To assess the PAD accuracy, results obtained with the developed device were compared to those obtained with the GC–MS reference method recommended by the UNODC [7]. Quantification with the GC–MS was done by determining the concentration of the phyto-cannabinoids CBD, CBN, Δ^8^-THC, and Δ^9^-THC and by using the summed concentration of the phyto-cannabinoids. The GC–MS method was performed using an Agilent GC–MS Triple quadrupole mass spectrometer (Agilent Technologies 7890A GC System equipped with an HP-5-MS column (30 m × 250 µm × 0.25 µm) and using calibration curve standards with a concentration of 0.5–10 mg L^−1^ of Δ^8^-THC, Δ^9^-THC, CBD, CBN, and an IS Δ^9^-THC-D3. (See Table S1 for the used GC–MS settings).

## Results and discussion

### Identification of the cannabis type (hemp or marijuana) using the 4-AP colorimetric reagent

The selection of 4-AP was not trivial, this reagent has some significant advantages compared to other colorimetric tests: i) the Duquenois-Levine test produces false positive results from components with a resorcinol group and an aliphatic chain, and is not useful for hemp and marijuana-type differentiation [12], and ii) the Fast Blue BB Salt (FBBB) produces an orange color for CBD-rich and red for THC rich cannabis, which can be hard to distinguish using the naked eye or smartphone-based applications [8].

On the other hand, the 4-AP test produces pink and blue colors resulting in a simple differentiation with the naked eye [11]. The 4-AP reagent was optimized for the rapid discrimination of THC- or CBD-rich cannabis samples, which is interesting because legal hemp and illegal cannabis cannot be distinguished by appearance or smell and, thus, requires analytical analysis. In this sense, the obtained results from plant samples were compared with those from the GC–MS reference method (see Table S2). A blue color corresponds to THC/CBN rich, and a pink color corresponds to CBD-rich samples, any other color is inconclusive. The color response perfectly matched the most abundant phyto-cannabinoids present in the samples (determined by GC–MS analysis). Correspondingly, the selectivity of the 4-AP color reagent was examined by testing 11 herbs, 3 tea sorts, and coffee (Table S3) to find possible misclassifications with free-THC samples. As observed in Table S3, only boldo (*peumus boldus*) gave a false positive result for THC/CBN, but after testing boldo with the quantification method for cannabis samples with the Fast Corinth V reagent, it did not provide a significant color response (< LOD), consequently, this mismatch classification is insignificant when the entire PAD procedure is applied.

### Optimization of developed PAD for total phyto-cannabinoid quantification

Several parameters that influence the phyto-cannabinoid quantification on the PAD were studied using Δ^8^-THC standards. Δ^8^-THC has been used as an analytical standard instead of Δ^9^-THC after assessing that the signal obtained from both compounds was statistically comparable. The studies were performed by establishing calibration curves for each assessed parameter discarding possible outliers.

The 4-AP test indicates the THC/CBD ratio present in a sample, but it cannot determine the percentage of THC available in the sample, thus the 4-AP test is not suitable for the quantification of phyto-cannabinoids [11]. Therefore, another colorimetric reagent is needed to determine the total phyto-cannabinoid content in cannabis samples. In terms of speed, color intensity, and selectivity, the Fast Corinth V reagent is superior compared to the D-L test and Fast Blue B test [7]. The Fast Corinth V reagent produces a pink color for all phyto-cannabinoids, and it was then selected for the quantification of phyto-cannabinoids in cannabis and oral fluids.

**Fig. 3:**
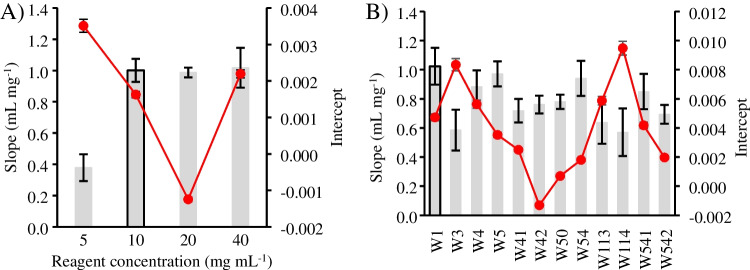
Physicochemical optimization of PAD design. Grey bars are corresponding to the slope values and red lines to the intercept values. A) Influence of reagent concentrations and B) filter paper nature on the sensitivity and intercept values. Experimental conditions: calibration range 0.005–0.10 mg mL^−1^ of Δ^8−^THC; reagent volume 1 µL; sample volume 0.5 µL; reading time 15 min. The error bars represent the standard error of the slope and a 5% percentage error of the intercept

First, the optimal Fast Corinth V reagent concentrations were studied in the 5–40 mg mL^−1^ range (see Fig. 3A). No significant differences (95% confidence level) in sensitivity and intercept were observed when the reagent was set at ≥ 10 mg mL^−1^. Therefore, 10 mg mL^−1^ was chosen as an optimal value due to the cost efficiency.

The type of filter paper used for the hydrophilic layer was evaluated by testing filter papers with different pore sizes, thicknesses, and manufacturing treatments (see details in Table S4). Several papers were examined, using the same experimental conditions as in the reagent study, to evaluate their influence on the signal response. Figure 3B shows that the highest sensitivity from the calibration curve slope was obtained for W1, W5, and W54, with no statistical difference between them (95% confidence level). Therefore, Whatman® nº 1, a qualitative paper with a pore diameter of 11 µm, was chosen due to its lower purchase cost.

### Features of the developed PAD method

#### Stability studies

**Fig. 4:**
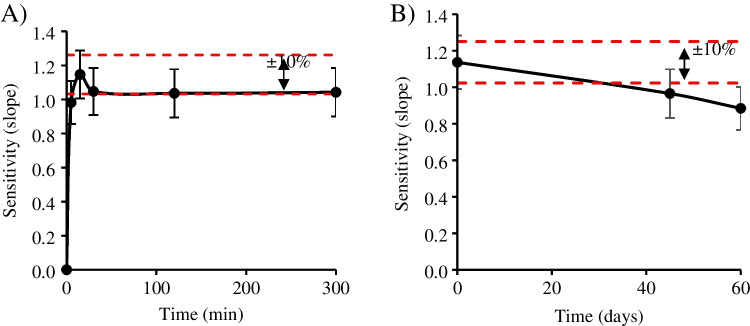
Stability of PAD cards (after injection) during storage at room temperature (approx. 25°C). A) Color stability low range (0–300 min). B) Color stability high range (0–60 days). Experimental conditions: calibration range 0.01–0.10 mg mL^−1^ of Δ^8^-THC; reagent volume 1 µL; sample volume 0.5 µL. The standard error of the calibration curve's slope is shown by error bars, and a 10% interval is included

The robustness of the present method was assessed in terms of color stability after the sampling process and the color is developed. To do so, the PAD was scanned over time after loading the standards in a low and high range (see Fig. 4). At low range (0–300 min), the sensitivity increased to its maximum at 1.15 mL mg^−1^ until 15 min when the equilibrium is reached (Fig. 4A). After this point, no significant differences (± 10%) were found in the following image captures over time (up to 5 h). In the extended time range (up to 60 days at 25 °C, 1 atm), Fig. 4B shows a statistical difference (± 10%) after 28 days after sampling. Therefore, the maximum time of PAD lifetime (in the mentioned conditions) is 1 month without loss of its initial performance, which favors not only the possible in-field sampling, but also the analysis of the suspicious sample up to 1 month later than the first analysis.

### Analytical characteristics of the developed PAD

Once the method was optimized, the features of the PAD method were assessed, including the linear range, the limit of detection (LOD), the limit of quantification (LOQ), precision, and stability (Table 1).

**Table 1:** Analytical performance of developed PAD for the quantification of phyto-cannabinoids

Analytical parameters, units	Value
Linear range, mg mL^−1^	0.01–0.1
Calibration equation^a^, A = slope ± SD mg mL^−1^; intercept ± SD	A = 0.993 ± 0.087 + 0.007 ± 0.001; R^2^ = 0.919 ± 0.009
LOD, mg L^−1^	3
LOQ, mg L^−1^	10
Precision intra-device, % RSD^b^	10; 9; 4 (for 0.02; 0.05; 0.08 mg mL^−1^, respectively)
Precision inter-device, % RSD^b^	15; 10; 8 (for 0.02; 0.05; 0.08 mg mL^−1^, respectively)
Repeatability intra-day, % RSD^a^	9
Repeatability inter-day, % RSD^a^	13
Color stability after sample addition^c^	1 month

LOD: limit of detection; LOQ: limit of quantification; precision; repeatability; color stability

(^a^ n = 3 calibration curves; ^b^ n = 4 calibration curves;^c^ storage after the sampling process without darkness at 25 °C, 1 atm)

Linearity was achieved in the linear range comprised between 0.01–0.10 mg mL^−1^ using Δ^8^-THC standards. The LOD and LOQ were calculated according to IUPAC recommendation [13], corresponding to a signal-to-noise ratio of 3 and 10, respectively. To this end, the standard deviation of ten measurements corresponding to the lowest concentration that can be reliably detected or quantified at these criteria was obtained, and divided by the slope of calibration curve. The precision was assessed by calculating the relative standard deviation (RSD) at three different concentration levels (n = 4 each). The repeatability was evaluated by calculation of the RSD of the obtained calibration curve slopes.

### Analytical responses of different phyto-cannabinoids

To determine the total content of phyto-cannabinoids in cannabis and oral fluid samples, the different sensitivity of THC, CBD, CBN and CBG using the Fast Corinth V colorimetric reaction was evaluated. It would be desirable to obtain the same sensitivity for the different phyto-cannabinoids. However, the colorimetric reagent is a simple way to analyze quickly any kind of sample and different phyto-cannabinoids present different sensitivity. In these cases, a compensation factor would be used to calculate the total phyto-cannabinoid content using the response of the main phyto-cannabinoid in each sample, obtained by the 4-AP reaction.

From the obtained results, CBD, CBN and CBG gave lower responses using the Fast Corinth V reagent than Δ^8^-THC (2.2x-, 1.3x, 2.8x, respectively). Thus, to obtain the total phyto-cannabinoid content in CBD rich samples, the obtained result would be multiplied by a factor of 2.2 (see “Analysis of cannabis samples” section).

### Assessment of potential matrix interferences

The reliability of the presented method was further assessed by analyzing several potential interferences of cannabis, namely, nicotine, caffeine, codeine, diazepam, cocaine, red tea, green tea, chai tea, coffee, tobacco, and two plant samples sprayed with synthetic phyto-cannabinoids (C1, C2). The solution of nicotine, caffeine, codeine, diazepam, and cocaine had a concentration of 0.05 mg mL^−1^ in methanol, and the tea, coffee, tobacco and synthetic phyto-cannabinoid extracts were obtained following the proposed cannabis sample preparation method (see “Sample preparation” section). A Δ^8^-THC standard with a 0.05 mg mL^−1^ concentration was analyzed as a colorimetric reference response in all cases. Table S5 summarizes the obtained signal and corresponding concentrations using a PAD. The obtained absorbance for all compounds was below the < LOD.

### Analysis of cannabis samples

The proposed PAD method was applied to the phyto-cannabinoid quantification in 18 different cannabis samples. The total phyto-cannabinoid contents obtained with the PAD by applying the normalization factor were compared with those obtained by GC–MS quantification as a reference method (Table 2). As observed, the founded concentration applying PAD technology is close to the reference value in all cases, with not significant differences between one another regardless the composition of the sample. The Pearson test (Figure S1) further confirms the non-statistical differences between the data obtained from GC–MS analysis compared to PAD technology (confidence level 95%). In this sense, the method allows the correct quantification, after the qualification, either the sample is CBD-, THC- or CBN-rich, which highly favors the suitability of this procedure.

**Table 2:** Comparison of total phyto-cannabinoid content in different cannabis samples using the reference GC–MS method and the proposed PAD method (n = 4)

Sample ID	Concentration GC–MS ± 5% interval (wt. %)	Concentration PAD (wt. %)^1^
CBD-rich		
M21-0001	19.1 ± 1.1	21 ± 2
M21-0002	16.8 ± 0.9	15.0 ± 1.5
M21-0004	19.1 ± 1.0	20 ± 2
THC-rich		
M21-0011	11.6 ± 0.6	12.0 ± 0.9
M21-0016	14.6 ± 0.7	16.6 ± 1.0
M21-0018	21.8 ± 1.6	20.3 ± 0.9
M21-0024	16.8 ± 0.8	14.8 ± 1.6
M21-0026	14.8 ± 1.0	13.1 ± 1.9
M21-0031	18.6 ± 1.0	17.7 ± 0.8
CBN-rich (degraded samples)		
M21-0012	12.1 ± 0.6	10.5 ± 1.2
M21-0014	6.5 ± 0.3	6.7 ± 0.7
M21-0017	18.6 ± 1.1	17.6 ± 1.2
M21-0020	20.4 ± 1.1	18 ± 3
M21-0023	15.3 ± 0.8	14.9 ± 1.3
M21-0029	16.9 ± 0.9	14.6 ± 3.5
M21-0030	14.8 ± 0.7	15 ± 2
M21-0032	20.3 ± 1.0	18.0 ± 0.9
M21-0033	6.3 ± 0.3	5.9 ± 0.6

^1^The PAD response has been corrected by multiplying by a factor (see “Analytical responses of different phyto-cannabinoids” section)

### Analysis of oral fluids

The proposed PAD method was also applied for the phyto-cannabinoids quantification in oral fluid samples, with sampling times direct, 1 h, up to 6 h after smoking. The saliva was selected as preferred sample due to the non-invasive sampling as well as the preservation of the analyte integrity at the studied conditions (direct analysis or storage in the freezer) [16, 17]. After the extraction with chloroform centrifugation and collection of the supernatant (see “Sample preparation” section), the PAD analysis was carried out. The phyto-cannabinoid concentration was determined with three independent samples. The total phyto-cannabinoid concentrations obtained with the PAD were compared with the GC–MS reference method (see Table 3). As previously observed, in the case of oral fluid analysis still match with the reference GC–MS procedure. Furthermore, it should be pointed out that the quantification can be performed in different times, allowing the analysis in a wide range of time. This fact, together with the color stability, encourages the applicability of PAD-method for in-field analysis.

**Table 3:** Comparison of total phyto-cannabinoid content in oral fluids after different sampling times using the reference GC–MS method and the proposed PAD procedure

Sampling time	Concentration GC–MS ± 5% interval (mg L^−1^)	Average concentration PAD ± SD (mg L^−1^)*
Direct	0.32 ± 0.02	0.30 ± 0.12
1 h	0.35 ± 0.03	0.31 ± 0.06
6 h	0.24 ± 0.02	0.24 ± 0.10

* n = 3 samples along with n = 4 assays

### Method comparison

The present PAD procedure was compared with other reported methods regarding phyto-cannabinoid analysis (Table S6). For instance, Pholsiri et al*.* has reported an electrochemical device using a cobalt phthalocyanine for Δ^9^-THC and CBD in cannabis oil [14]. They achieved a linear range of 0.01 – 0.5 mg mL^−1^, LOD of 0.003 mg mL^−1^, RSD < 5%, and stability of 1 month, most of which are comparable to those reported here. However, this method requires complicated equipment and skilled handlers.

Regarding a thin-layer chromatography (TLC) approach for the qualification of phyto-cannabinoids using the Fast Blue B reagent [15], The TLC method showed good color determination using RGB codes. However, the technique requires higher volumes, is time-consuming, is not able to quantify the analytes, and TLC systems are more sensitive to humidity, which causes lower reproducibility [16].

On the other hand, the proposed method achieved worse LODs and linear range compared to fluorescence, electrophoresis, and chemiluminescence techniques [17–19]. However, these methods require sophisticated non-portable equipment and more analysis time, resulting in unsuitable for non-skilled operators.

Lastly, Jornet-Martínez, N. et al*.* have proposed a method based on Fast Blue B reagent immobilized into polydimethylsiloxane resin. Although it is an interesting and useful approach, the reported stabilities are only up to 10 days [9], being this characteristic somehow limiting for the point-of-care (POC) analysis.

Furthermore, comparing the PAD cards to traditional techniques (spectrophotometry, immunoassay, chromatography, and mass spectrometry) [20–22], the proposed method offers significant advantages such as portability, non-expensiveness, and ease to use and also being in agreement with the Green Analytical Chemistry principles (Table S7) [23].

### Green assessment

To further support the suitability of the developed method, its greenness has been evaluated by using “AGREE—Analytical GREEnness Metric Approach and Software” [24] (Table 4) and compared to GC–MS as reference method. As observed, the 12 principles were evaluated for both approaches. At first sight, the portability of the proposed PAD affects to the principle 1 and 3, getting a better score than non-portable GC–MS. Furthermore, the possibility to perform 75 samples per hour (24-reaction positions per PAD, 4 PAD every 15 min) together with the low waste generation increase the suitability of the present method for drug screening purposes. Although similar reagents are being used, the large amount reagents to inject them as well as the energy consumption of the GC system make this alternative worse from the green point of view. In any case, the proposed method is not claiming to replace other confirmation techniques, such as GC–MS. However, it could be a promising alternative due to its speed and simplicity as a screening technique.

**Table 4:** Evaluation of the present method from the green point of view

Principles	Score	
	PAD analysis	GC–MS analysis
1) Direct analytical techniques	At-line analysis (0.60)	Off-line analysis (0.48)
2) Minimal sample size	0.2 g/mL of sample (0.88)	0.2 g/mL of sample (0.88)
3) In Situ measurements	At-line (0.33)	Off-line (0.0)
4) Integration of analytical processes	Less than 3 sample pretreatment steps (1.0)	Less than 3 sample pretreatment steps (1.0)
5) Automation and miniaturization	Manual / non-sample prep or miniaturized (0.5)	Manual / non-sample prep or miniaturized (0.5)
6) Derivatization	None (1.0)	None (1.0)
7) Generation of a large waste	5 g of waste (0.48)	5 g and 15 mL of waste (0.29)
8) Multianalyte or multiparameter methods	3 Analytes / 75 analyses per hour (1.0)	3 Analytes / 4 analyses per hour (0.55)
9) The use of energy	Non-instrumental detection (1.0)	GC–MS (0.0)
10) Reagent source	Non bio-based (0.0)	Non bio-based (0.0)
11) Toxicity of reagents	Yes, but low amount (0.8)	Yes, but 7.5 mL (0.24)
12) The safety of the operator	Highly flammable (0.8)	Highly flammable (0.8)
FINAL SCORE	0.7	0.48
Pictogram		

## Conclusions

A cost-effective (€0.10 per PAD) and portable PAD (< 5 g) method for the determination of phyto-cannabinoids in cannabis and oral fluids using colorimetry has been described in this paper. The designed PAD was based on simple stationery materials assuring its use by non-specialized personnel. It serves as two analytical tools, the 4-AP reagent for qualification of THC/CBD in plant samples and the quantification of phyto-cannabinoids in plant and oral fluids by using the Fast Corinth V reagent, being both approaches effective and reliable. The developed microfluidic device offers a simple and fast alternative for POC analysis since it doesn’t require external pumps neither expensive equipment. Although the 4-AP test has some disadvantages, such as inconclusive results when the THC/CBD ratio is within the 0.3–3 range, or the impossibility to determine if the THC concentration is below a certain value, it is the best option compared to other candidates (*e.g.*, Duquenois-Levine test and FBBB) as explained in “Identification of the cannabis type (hemp or marijuana) using the 4-AP colorimetric reagent” section. The proposed PAD method showed reduced linear range (0.01 – 0.1 mg mL^−1^), but very good LOD (0.003 mg mL^−1^) and suitable precision (RSD < 10%). The real applicability was assessed with potential interferents present in samples, being negligible when the two colorimetric reagents are used. Then, the content in plant and oral fluids was investigated and compared with PAD, obtaining acceptable results considering the simplicity of the proposed method. To the best of our knowledge, this is the first study that applied and demonstrated a paper-based method for the qualification and quantification of phyto-cannabinoids in plant and oral fluids using colorimetry. Overall, PADs have a lot of potential in screening applications (Table S7), especially in drug monitoring due to the high-throughput (75 samples per hour). The application scenarios are many including herb product quality control, labelling monitoring, police drug test as well as sport drug controls, among others. It can serve as a guide for future studies in analytical chemistry as an affordable tool and in (bio)analysis for diagnosis, although aspects such as LOD as well as selectivity should be further studied in future contributions.

### Supplementary Information

Below is the link to the electronic supplementary material.

Supplementary file1 (DOCX 41 KB)
