# Supplementary material for: Miniaturized paper-based analytical device for the portable analysis of phyto-cannabinoids in plant and oral fluids
Source: Anal Bioanal Chem. 2023 Nov 4;416(1):255–64. doi: 10.1007/s00216-023-05013-x (PMC10758360; doi:10.1007/s00216-023-05013-x)
Supplement: Supplementary file 1 — Supplementary file1 (DOCX 41 KB) [file 216_2023_5013_MOESM1_ESM.docx]

**ELECTRONIC SUPPLEMENTARY MATERIAL**

**Miniaturized paper-based analytical device for the portable analysis of phyto-cannabinoids in plant and oral fluids**

Dymphy Houtzager, Sergio Armenta, José Manuel Herrero-Martínez, Héctor Martínez-Pérez-Cejuela*

*Department of Analytical Chemistry, University of Valencia, Dr. Moliner 50, 46100, Burjassot, Valencia, Spain*

*Corresponding authors: Héctor Martínez-Pérez-Cejuela

Tel.: +34 9635 44062

e-mail: [Hector.Martinez-Perez@uv.es](mailto:sergio.armenta@uv.es)

**Table of contents**

**Page S1.** GC-MS settings (Table S1)

**Page S2.** Qualitative assessment (Table S2)

**Page S3.** Interference studies for qualification (Table S3)

**Page S4.** Characteristics of used papers (Table S4)

**Page S5.** Interference studies for quantification (Table S5)

**Page S6.** Table comparison (Table S6)

**Page S7.** PAD features and advantages (Table S7)

**Page S8.** Pearson plot of phyto-cannabinoid quantification (Figure S1)

**Table S1.** GC-MS settings reference method.

| Parameter | Value |
| --- | --- |
| Carrier gas | Helium |
| Oven temperature (start) | 150 °C min^-1^ |
| First ramp | 10 °C min^-1^ to 250°C |
| Oven temperature (end) | 250 °C per 10 min |
| Flow | 1 mL min^-1^ |
| Inlet temperature | 250 °C |
| Inlet mode | Splitless |
| Solvent delay | 5 min |
| Ion source temperature | 276 °C |
| Quadrupole temperature | 150 °C |

**Table S2.** Cannabis samples with the most abundant cannabinoid present and corresponding color observed with the 4-AP color reagent.

| Cannabis sample | Most abundant cannabinoid | Observed colour^1^ |
| --- | --- | --- |
| 0001 | CBD | Pink |
| 0002 | CBD | Pink |
| 0006 | Mix CBD/Δ^9^-THC/CBN | Purple |
| 0012 | CBN | Blue |
| 0014 | CBN | Blue |
| 0017 | CBN | Blue |
| 0018 | Mix Δ^8^-THC/Δ^9^-THC/CBN | Blue |
| 0020 | CBN | Blue |
| 0023 | CBN | Blue |
| 0024 | Mix Δ^8^-THC/Δ^9^-THC/CBN | Blue |
| 0025 | CBN | Blue |
| 0026 | Mix Δ^8^-THC/Δ^9^-THC/CBN | Blue |
| 0029 | CBN/Δ^9^-THC | Blue |

^1^Blue color corresponds to THC/CBN rich, the pink color corresponds to CBD rich, any other color is inconclusive.

**Table S3.** Interferences from relevant compounds of cannabis with the 4-AP reagent using the developed PAD method. Reagent A & B volume 1 µL; sample volume 0.5 µL; reading time 5 min.

| Herb | Result^1^ |
| --- | --- |
| Salvia lavandulifolia | no color |
| Boldo (peumus boldus) | Blue color |
| English lavender (Lavandula angustifolia) | no color |
| Thymus hyemalis | other color |
| Sen hojas (cassia angusfolia) | no color |
| Illicium verum | other color |
| Papaver rhoeas | no color |
| Citrus aurantium | other color |
| Equisetum arvense | no color |
| Matricaria chamomilla | other color |
| Taraxacum officinale | other color |
| Red tea | other color |
| Green tea | other color |
| Chai tea | other color |
| Coffee | other color |

^1^Other color: Reaction produced a color different than pink and blue.

**Table S4.** Different properties of the Whatman® filter papers used during this work.

| Whatman number | Paper type | Pore size (µm) | Thickness (mm) |
| --- | --- | --- | --- |
| W1 | Qualitative | 11 | 0.18 |
| W3 | Qualitative | 6 | 0.39 |
| W4 | Qualitative | 20-25 | 0.21 |
| W5 | Qualitative | 2.5 | 0.20 |
| W41 | Ashless quantitative | 20-25 | 0.22 |
| W42 | Ashless quantitative | 2.5 | 0.20 |
| W50 | Hardened Low Ash | 2.7 | 0.12 |
| W54 | Hardened Low Ash | 20-25 | 0.19 |
| W113 | General Purpose and Wet-Strengthened | 30 | 0.42 |
| W114 | General Purpose and Wet-Strengthened | 25 | 0.19 |
| W541 | Hardened Ashless | 20-25 | 0.16 |
| W542 | Hardened Ashless | 2.7 | 0.15 |

**Table S5.** Interferences from relevant compounds of cannabis with the Fast Corinth V reagent using the developed PAD method. Drugs concentration 0.05 mg mL^-1^; reagent volume 1 µL; sample volume 0.5 µL; reading time 15 min.

| Component | Pseudo-absorbance | Concentration THC  (mg mL^-1^) |
| --- | --- | --- |
| *Drugs of interest* | | |
| Nicotine | 0.004 | <LOD |
| Caffeine | 0.003 | <LOD |
| Codeine | 0.002 | <LOD |
| Diazepam | -0.003 | <LOD |
| Cocaine | 0.000 | <LOD |
| *Solid samples of interest* | | |
| Synthetic cannabinoid 1 | 0.006 | <LOD |
| Synthetic cannabinoid 2 | -0.008 | <LOD |
| Chai tea | 0.002 | <LOD |
| Coffee | -0.008 | <LOD |
| Tobacco | -0.005 | <LOD |
| Red tea | -0.002 | <LOD |
| Green tea | -0.003 | <LOD |

**Table S6.** Comparison between the developed PAD procedure and similar methods reported in the literature.

| Analytes | Detection | Support material | Sample matrix | Detection time (min)^a^ | RSD (%) | LODs (mg L^-1^) | Ref. |
| --- | --- | --- | --- | --- | --- | --- | --- |
| Δ^9^-THC, CBD and CBN | Colorimetric | Polydimethylsiloxane | Cannabis plant | 1 | ≤9 | 3.7 | [9] |
| Δ^9^-THC and CBD | Electrochemical | Paper (wax printing) | Cannabis oil | 15 | <5 | 3 | [14] |
| Δ^9^-THC, CBD and CBN | Colorimetric | Aluminium thin layer – Silica | Cannabis plant | - | - | Not determined | [15] |
| Δ^9^-THC, CBD and CBN | Colorimetric | Paper (stationery material) | Oral fluids and cannabis plant | 15 | <15 | 3 | This work |

**Table S7.** PAD features and advantages from the use of the present method.

| PAD features^1^ | |
| --- | --- |
| Low cost | < 0.1 Euro cents |
| Lightweight | < 5 g |
| Required materials | Laminating pouch, paper filter, laminator |
| Detection system | Scan/smartphone |
| Throughput | 75 samples per hour |
| Low reagent/sample consumption  Qualification | 7.2 µg 4-AP  40.7 µL EtOH  0.12 µL HCl 2 M  0.72 mg NaOH  7.2 µL H_2_O  24 µL sample |
| Low reagent/sample consumption  Quantification | 240 µg Fast Corinth V  24 µL H_2_O  12-24 µL sample |
| Type of samples | Liquids (direct)  Solid (after extraction) |

^1^For a one PAD card (24 analysis positions)

**Figure S1.** Pearson plot of phyto-cannabinoid quantification using the developed PAD method and the GC-MS reference method; the concentration points are the average from 3 different replicates.
